# Genetic and Molecular Mechanisms of Synaptic Plasticity in Perioperative Delirium: From Neuroinflammation to Circuit Dysfunction and Recovery

**DOI:** 10.3390/genes17101315

**Published:** 2026-10-21

**Authors:** Zheng Liu, Tong Wang, Xinran He, Siyuan Song

**Affiliations:** 1 Department of Pathology, The University of Texas MD Anderson Cancer Center, Houston, TX 77030, USA; zhengliu.jan@gmail.com; 2 Department of Biology, Duke University, Durham, NC 27708, USA; wangtongnelly@gmail.com; 3 Department of Anesthesiology, Chobanian & Avedisian School of Medicine, Boston University, Boston, MA 02118, USA; xinran.he@bmc.org; 4 Department of Anesthesiology, Montefiore Medical Center, Albert Einstein College of Medicine, Bronx, NY 10467, USA

**Keywords:** postoperative delirium, synaptic plasticity, APOE, microglia, neuroinflammation, epigenetics

## Abstract

Perioperative delirium is an acute disturbance of attention and awareness that arises when surgical stress interacts with limited brain reserve. Synaptic dysfunction provides a plausible connection between genetic susceptibility, neuroimmune responses and fluctuating network activity, but the strength of evidence differs substantially across these levels. This narrative review examines human genetic and molecular studies alongside experimental investigations of synaptic plasticity, with emphasis on recent primary research. Large association studies now support an association between *APOE* ε4 and delirium susceptibility, whereas blood transcriptomics and brain tissue profiling implicate complement regulation, immune responses and glial cell states. Experimental studies identify mechanisms involving complement-dependent synaptic engulfment, astrocytic cholesterol and glucose support, glutamate handling, mitochondrial stress and epigenetic regulation of BDNF–ARC signaling. These findings suggest that reversible changes in synaptic efficacy and slower structural remodeling can coexist, without requiring synapse loss to explain every delirium episode. Human neurogranin, neurofilament and electrophysiological studies also reveal important negative or context-dependent findings. Translation therefore requires separation of preoperative vulnerability from acute disease activity, direct assessment of attention and arousal, and evidence that a molecular intervention improves clinically meaningful outcomes. The phase 2 trial of the APOE mimetic CN-105 illustrates the distinction between mechanistic plausibility and clinical efficacy, as its benefit for delirium prevention remains unproven. A cell-specific, temporally resolved approach is needed to determine which synaptic mechanisms are causal, reversible and therapeutically accessible in human perioperative delirium.

## 1. Introduction

Perioperative delirium presents a mechanistic problem that a single inflammatory marker or neurotransmitter hypothesis cannot resolve. Attention may deteriorate over hours, improve transiently and worsen again, while recovery of everyday cognition may take substantially longer. Clinical assessment must therefore establish an acute change from baseline and characterize its fluctuations rather than infer delirium from a low cognitive screening score. The perioperative nomenclature consensus separates postoperative delirium from delayed neurocognitive recovery and postoperative neurocognitive disorder [1]. Throughout this review, perioperative delirium is used as a broad contextual term, whereas postoperative delirium (POD) refers specifically to delirium occurring after surgery. Postoperative cognitive impairment in experimental models is not considered equivalent to clinically diagnosed POD. This distinction is central to interpreting molecular experiments: impaired fear conditioning after surgery does not, by itself, establish an animal equivalent of human delirium.

The clinical consequences extend beyond the immediate admission. In the Successful Aging after Elective Surgery cohort, delirium was associated with a steeper cognitive decline over six years [2]. A subsequent analysis found that recurrent hospitalizations explained little of this association [3]. These observations justify studying mechanisms of incomplete recovery, but they do not establish that delirium invariably causes neurodegeneration. Pre-existing pathology, perioperative injury and the consequences of the episode may each contribute. The biological question is consequently twofold: why does a particular brain become acutely dysfunctional, and which processes determine whether its function returns to the previous trajectory?

Synapses are a useful point of convergence because their operation depends on neuronal receptors, glial support, vascular supply and activity-dependent gene expression. A change in any of these systems can alter information processing without immediately destroying neurons. Recent work has substantially improved the human evidence base. Genome-wide studies implicate the *APOE* region in postoperative and broader delirium susceptibility [4,5]; blood transcriptomics identifies immune and complement-related signatures [6,7]; and surgical brain tissue provides access to cell-type-resolved molecular differences [8]. These discoveries should be connected to experimental synaptic mechanisms while preserving the distinction between association, pathway perturbation and clinical efficacy.

**Figure 1:**
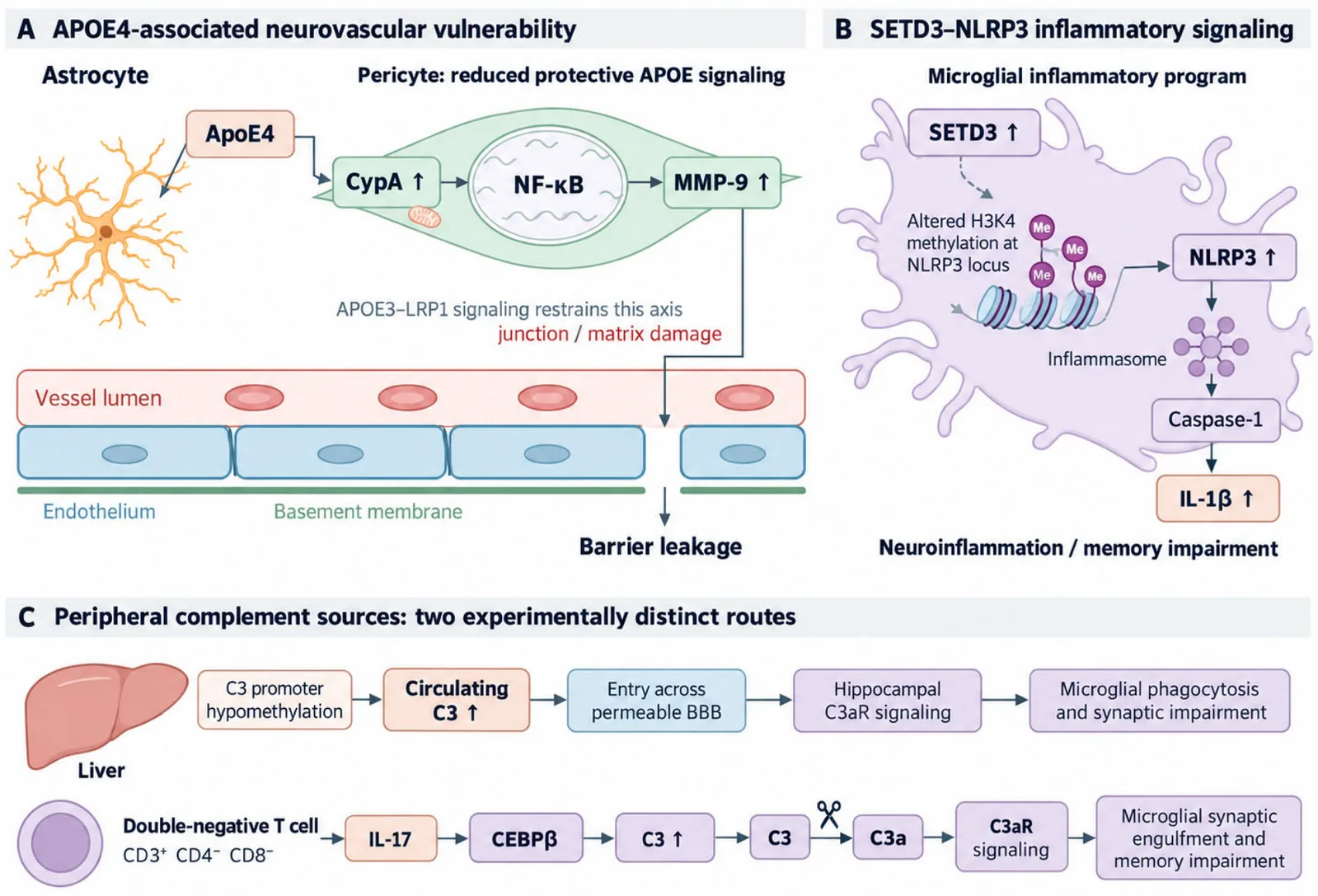
**Genetic susceptibility and perioperative immune signaling across compartments.** (**A**) APOE4-associated neurovascular vulnerability involves CypA–NF-κB–MMP-9 signaling in nonsurgical models [9,10]; human *APOE*-associated findings provide susceptibility context [11]. (**B**) SETD3 manipulation was associated with altered H3K4 methylation near the *NLRP3* locus and inflammatory signaling [12]. Direct H3K4 methylation by SETD3 was not established. (**C**) Liver-derived C3 and IL-17/C/EBPβ-associated C3 induction represent distinct experimental pathways [13,14]. C3 cleavage generates C3a, the ligand for C3aR; the cleavage step is schematic, and its timing and location were not directly established in these studies. BBB, blood–brain barrier; CypA, cyclophilin A; POD, postoperative delirium. Colors distinguish the labeled cells, compartments, and pathway components and do not encode quantitative values. Connecting arrows show the proposed direction of signaling or transport; the dashed arrow indicates an indirect association rather than established direct methyltransferase activity. Upward arrows indicate increases, and the scissors denote C3 cleavage. The breaks in the endothelial layer depict barrier disruption. CD3+, CD4−, and CD8− denote marker-positive and marker-negative cells, respectively.

This review develops that connection around genetic and epigenetic susceptibility, neurovascular and glial signaling, synaptic function, network disruption and recovery. Figure 1 resolves selected genetic, vascular and inflammatory pathways across cellular compartments. We emphasize that transient impairment of transmission, maladaptive plasticity and structural synapse elimination are different biological events. They can interact, but current human evidence does not support treating them as interchangeable explanations for every delirium phenotype.

### Scope and Evidence Selection

A targeted narrative search of PubMed-indexed literature, Europe PMC records, and relevant publisher websites was conducted through 23 September 2026. Representative search terms combined “delirium,” “postoperative delirium,” or “perioperative neurocognitive disorders” with terms including “genetics,” “APOE,” “DNA methylation,” “transcriptomics,” “complement,” “microglia,” “astrocytes,” “synaptic plasticity,” “blood–brain barrier,” and “electrophysiology.” Studies were selected for detailed full-text review based on their relevance to perioperative delirium, clarity of clinical phenotype, experimental methodology, and contribution to molecular or synaptic mechanisms. Human studies with clearly defined delirium outcomes and experimental studies incorporating pathway perturbation or functional measurements were prioritized. Earlier foundational studies and nonsurgical models were included when necessary to provide mechanistic context. This was a narrative rather than systematic review; no formal risk-of-bias assessment or quantitative synthesis was performed.

## 2. Genetic Susceptibility and Epigenetic Regulation

### 2.1. APOE and the Limits of Genetic Inference

The strongest recent genetic evidence concerns *APOE*. Armstrong and colleagues analyzed 1016 postoperative delirium cases and 139,148 surgical controls in UK Biobank. The lead variant was rs429358, and gene-level signals involved *APOE*, *TOMM40*, *APOC1* and *PVRL2* within the same chromosome 19 region. Relative to noncarriers, one and two *APOE ε4* alleles were associated with odds ratios of 1.75 and 4.19, respectively, after adjustment for age and sex [4]. These are susceptibility estimates from a coded phenotype, not estimates of an individual patient’s inevitable outcome. Moreover, several associated genes in a linkage-disequilibrium block do not constitute proof of several independent causal pathways.

Evidence extends beyond surgical cohorts. Raptis and colleagues studied 11,931 delirium cases within a total sample of 1,059,130 individuals and identified a strong *APOE* association. Their multi-trait analysis with Alzheimer disease increased the number of implicated loci, while integrated plasma proteomics suggested additional candidate biology [5]. The broader phenotype improves statistical power but also combines different precipitants and clinical contexts. A separate surgical biobank study of 33,526 patients found an association between *APOE* ε4 and delirium, with an odds ratio of 1.32; most participants had European ancestry [15]. Differences in ascertainment, ancestry, comorbidity and exposure windows help explain why effect sizes should not be directly compared as if they came from one harmonized experiment.

The biological interpretation of *APOE* is broader than amyloid deposition. In humans, carriage of the *APOE* ε4 allele has been associated with hippocampal and medial temporal blood–brain barrier abnormalities and pericyte injury markers, including in cognitively unimpaired carriers [11]. Human Alzheimer brain profiling and induced pluripotent stem cell-derived microglia also identify an APOE4-associated lipid-droplet state involving ACSL1 [16]. These observations offer plausible routes to reduced vascular, metabolic or glial reserve. They are not demonstrations that the same route mediates the *APOE*–delirium association after surgery.

The neurovascular hypothesis can be specified at the level of individual cells. In APOE mouse models, inadequate suppression of pericyte cyclophilin A permits NF-κB activation and MMP-9-dependent barrier damage; intervention at this pathway improved vascular integrity [9]. Aged APOE4/5xFAD experiments extended the finding to advanced disease, with protection independent of a reduction in amyloid-β [10]. Multi-omics profiling also placed endothelial and pericyte abnormalities before later synaptic dysfunction in APOE4 mice [17]. These experiments provide intervention-supported evidence for a potential vascular mechanism.

The apoE4 protein can also act outside neurons. Liver-restricted apoE4 expression compromised cerebrovascular function and cognition in experimental systems [18], while border-associated macrophages were identified as both a source and an effector of apoE4-dependent oxidative neurovascular injury [19]. Together, the studies justify separating circulating apoE, pericyte signaling and interface macrophages when testing the vascular component of genetic susceptibility.

Genotype should therefore be treated as a stable susceptibility layer. A useful perioperative model would ask whether it adds calibrated predictive information beyond cognition, frailty, sensory impairment and surgical context, and whether that information changes a decision. A genetic association alone does not justify routine *APOE* testing or an APOE-directed intervention. Analyses of Alzheimer polygenic risk should explicitly separate the dominant *APOE* region from the remaining score and evaluate portability across ancestries. Multi-trait discovery and Mendelian-randomization prioritization are valuable starting points, but fine-mapping, colocalization and experimentally validated cell-specific effects remain necessary before assigning a causal target.

### 2.2. Dynamic Methylation and Transcriptional Responses

Epigenetic studies address a different question: how baseline vulnerability and an acute exposure are reflected in gene regulation. A 2024 investigation compared blood DNA methylation across four independent delirium cohorts and reported directional replication of several previously identified CpG signals [20]. Earlier neurosurgical studies found immune-related enrichment in perioperative blood and explored brain, blood, saliva and buccal tissues from the same participants [21,22]. The latter study did not identify genome-wide significant CpGs, and overlap across tissues was limited. Pathway enrichment based on nominal signals can generate hypotheses; it does not establish a validated molecular diagnostic test or a blood surrogate for brain chromatin.

Timing is particularly informative. In a small gastrointestinal surgical cohort, immune-related methylation differences were more evident in preoperative-to-day-1 comparisons than in preoperative-to-day-3 comparisons [23]. A 2026 hip-fracture investigation, analyzing 65 participants after propensity-based selection, similarly found early immune and inflammatory pathway enrichment that became less prominent in later comparisons [24]. These findings are consistent with a transient perioperative response. However, blood cell composition, infection, medication exposure and surgical injury can also change methylation estimates. A dynamic blood signature should not be described as a stable inherited predisposition, and postoperative differences cannot automatically be interpreted as causes of an episode that has already begun.

Larger transcriptional studies reinforce this distinction. In 599 older surgical patients, preoperative blood expression did not significantly distinguish those who subsequently developed delirium, whereas postoperative baseline-corrected expression implicated immune responses, RNA metabolism and platelet-related processes [7]. Conversely, a nested MINDDS analysis found lower preoperative expression of the complement regulators *C4BPA* and *CD55* in patients developing delirium, with *C4BPA* evaluated by quantitative PCR in a larger cohort [6]. These results need not be contradictory. They interrogate different populations, assays and biological states, and they distinguish a specific candidate signature from a broad transcriptome-wide comparison.

For mechanistic interpretation, the regulatory chain must be demonstrated rather than assumed: an epigenetic alteration should affect transcription in a specified cell type, change a measurable synaptic process and contribute to an attention or arousal phenotype. In a recent aged-mouse model combining anesthesia, surgery and intensive-care-like stress, reduced BDNF–ARC signaling accompanied histone hypoacetylation, altered cytosine modifications and impaired attention; histone deacetylase inhibition attenuated several abnormalities [25]. This is a useful intervention-based hypothesis, but the broad actions of chromatin-modifying drugs prevent attribution to one gene without more selective perturbation.

A second mechanistically distinct epigenetic route involves inflammatory gene regulation. In an anesthesia/surgery model, SETD3 upregulation was associated with microglial reactivity and activation of NLRP3–caspase-1–IL-1β signaling. SETD3 knockdown reduced H3K4 methylation near the *NLRP3* transcription start site in cultured cells and attenuated postoperative neuroinflammatory and cognitive abnormalities [12]. These findings link SETD3 manipulation to changes in promoter-associated histone methylation but do not establish direct H3K4 methylation by SETD3. Promoter-associated histone methylation, DNA cytosine modification and histone acetylation should therefore remain separate variables. A change in one mark cannot be used as evidence that another mark or another cell population was altered [12,25].

### 2.3. What Human Brain Tissue Adds

Ishii and colleagues profiled brain tissue from 18 patients undergoing epilepsy surgery, including nine who later developed delirium after resection. Microglial inflammatory signatures and astrocytic pathway changes provided direct evidence that relevant cell states can be studied in human surgical tissue [8]. The interpretation requires restraint: tissue came from heterogeneous regions of an epilepsy population, delirium was classified retrospectively, and the specimen was obtained during surgery rather than sampled serially through the episode. No methylation sites reached genome-wide significance. The study therefore supplies an unusually valuable, but small and context-specific, link between brain biology and subsequent delirium. It does not establish a universal delirium cell state or prove that methylation changes drive the clinical syndrome. Table 1 summarizes the human findings most relevant to this distinction.

## 3. Neurovascular and Glial Mechanisms

### 3.1. Systemic Inflammation and the Brain Interfaces

Surgery produces a coordinated immune response, and the relevant question is why its effect on brain function differs between patients. Experimental work placed TNF upstream of an IL-1-dependent inflammatory cascade after surgery and showed that peripheral TNF blockade could protect postoperative cognition in mice [28]. Other studies distinguish reversible working-memory dysfunction induced by systemic inflammation from IL-1-dependent neuronal injury in a brain with pre-existing pathology [29]. This separation is important: a transient delirium-like state and a mechanism contributing to persistent tissue injury can share an inflammatory trigger without being the same process.

The human signal is heterogeneous. In a prospective multicenter critical-illness cohort, IL-6, IL-8, IL-10 and other inflammatory measures were associated with next-day delirium, whereas IL-1β was not [30]. These data support immune involvement but are not specific to postoperative delirium, and an association of an anti-inflammatory cytokine such as IL-10 may reflect response magnitude rather than a directly injurious action. Concentration alone cannot identify the signaling compartment, receptor configuration or direction of causality. Biomarkers should be interpreted together with infection, organ failure, sedation and the time of clinical assessment.

The vascular and cerebrospinal fluid interfaces may shape how systemic signals reach neural circuits. A perioperative cohort linked changes in the CSF-to-plasma albumin ratio and plasma S100B to delirium severity, although paired CSF analyses were small [31]. More recent work found that CSF soluble IL-6 receptor and a surrogate of IL-6 trans-signaling correlated with delirium severity in an exploratory cohort [32]. Such observations motivate receptor-specific studies rather than indiscriminate suppression of all IL-6 activity. They also require careful terminology: the albumin quotient reflects blood–CSF barrier function and CSF dynamics and is not a spatially resolved measurement of parenchymal endothelial permeability.

Not all central inflammatory mediators appear to arise from passive leakage. A combined hip-fracture and experimental investigation found that the relationship between chemokines, barrier permeability and delirium differed among molecules; mouse studies demonstrated local synthesis at the choroid plexus and microvasculature [33]. Another human metabolomic study linked phosphatidylcholine compartmental relationships to albumin-quotient differences without resolving whether altered production or transport was responsible [34]. These results argue for measuring paired compartments and evaluating barrier transport, local synthesis and clearance separately. A circulating endothelial-cell signature associated with delirium in postoperative critical illness offers a further vascular signal, but sepsis-related biology limits direct generalization to uncomplicated elective surgery [35].

A defined damage-sensing pathway also connects perioperative inflammation to trophic failure. In aged male rats exposed to surgery and morphine, central TLR4 antagonism prevented or reversed persistent postoperative memory impairment and restored synaptic proteins, mature BDNF and TrkB signaling [36]. The experiment supports TLR4-dependent disruption of plasticity in that exposure setting; it does not identify all perioperative opioids or all TLR4 activity as uniformly harmful. In parallel, a longitudinal human surgical cohort linked early IL-6 and phosphorylated-tau trajectories to delirium and later neurocognitive outcomes [37]. Human sampling can therefore test the timing predicted by experimental pathways, while remaining unable on its own to prove receptor-level causation.

### 3.2. Complement and Selective Synaptic Engulfment

Complement provides a mechanistic link between immune signaling and circuit structure. Foundational studies established roles for complement in developmental synapse elimination and activity-dependent microglial pruning [38,39]. In Alzheimer mouse models, complement-dependent microglial mechanisms can contribute to early synapse loss [40]. These experiments establish biological capability. They do not show that an elevated circulating complement concentration is evidence of active synaptic pruning in a postoperative patient.

The recent perioperative studies are more informative because they combine molecular localization with perturbation. In a fracture-surgery mouse model, C3, but not C1q, increased in association with presynaptic elements. Global *C3ar1* knockout or pharmacological inhibition of C3aR–STAT3 signaling attenuated excitatory synapse loss and memory impairment [41]. Although microglial involvement was supported by complementary cellular experiments, the global knockout does not establish microglia-specific causality in vivo. In another study, postoperative C1q colocalized with both excitatory and inhibitory synaptic markers, and microglial C1q depletion or a neutralizing antibody attenuated synaptic and behavioral abnormalities [42]. These different findings should be retained rather than merged into one compulsory linear cascade. Region, age, operation, sampling time and the synaptic compartment examined can determine which complement component is most important.

Peripheral organs may contribute additional complement sources. A study combining patient observations with experimental work implicated liver-derived C3, postoperative barrier disruption and hippocampal C3aR-related microglial phagocytosis [13]. An adult-mouse abdominal surgery model linked IL-17-producing double-negative T cells to C/EBPβ-dependent C3 expression and synaptic engulfment [14]. C3aR signaling and CR3-mediated recognition of opsonized material are distinct mechanisms, making receptor-specific evidence essential for interpreting these findings [39,41].

Human *C4BPA* and *CD55* findings bring this field closer to a clinical question: does inadequate complement regulation identify a subgroup with an exaggerated perioperative response [6]? Answering it requires paired measurements of gene expression, protein abundance and complement activity, ideally with a central or neurophysiological correlate. It also requires showing that the association is not explained by leukocyte composition. Complement inhibition could alter host defense and tissue repair as well as synaptic engulfment. Cell type, timing and duration of treatment therefore need to be established before a systemic anti-complement strategy can be inferred from mouse rescue experiments [13,41,42].

Human-cell systems offer an intermediate test of complement production. In a defined pluripotent-stem-cell-derived neuron–astrocyte–microglia tri-culture, reciprocal glial signaling contributed to C3 production in an Alzheimer-related genetic context [43]. This result makes cell-source assignment experimentally tractable, but it does not substitute for surgical exposure or a delirium phenotype. It also cautions against drawing a single arrow from blood C3 directly to synapse loss without testing local synthesis and the relevant receptor [13,41,43].

### 3.3. Microglia and Astrocytes Are Partners with Multiple Functions

Microglial activation is an insufficient description of the mechanism. Surveillance, phagocytosis, cytokine production, lipid handling and metabolic adaptation can change independently [16,44,45]. A 2024 imaging and ultrastructural study showed that microglial processes can enter inhibitory synaptic clefts during isoflurane anesthesia and facilitate post-anesthetic neuronal activity by shielding GABAergic input [44]. These findings highlight the context-dependent functions of microglia, including potentially protective responses that could be lost with indiscriminate suppression [44,46].

Astrocytes can alter the accessibility and function of synapses. In a developmental sensory-remodeling model, microglial Wnt signaling reduced astrocytic contact before microglial synapse removal [47]. In a perioperative mouse model, astrocytic phagocytosis in the medial prefrontal cortex was associated with loss of hippocampal inputs and impaired memory consolidation [48]. These experiments identify distinct routes to circuit remodeling, not a single interchangeable category of glial pruning. They also show why bulk expression of an inflammatory gene cannot identify the cellular effector or the affected projection.

Recent cell atlases broaden the view beyond a microglia–neuron pair. Single-cell profiling of aged postoperative mouse hippocampus implicated altered metabolism, autophagy, circadian processes and communication among neurons, microglia, astrocytes and oligodendrocytes [45]. Ligand–receptor inference from such data should guide experiments, not substitute for evidence of ligand release, receptor engagement or physiological necessity. Human CSF YKL-40 and sTREM2 studies also suggest that glial signals depend on dementia status and other clinical context [49,50]. Neither protein provides a direct readout of synaptic engulfment or establishes that increasing or decreasing the corresponding pathway would benefit the patient.

Homeostatic microglial signaling is concrete rather than merely a caveat. In nonsurgical mouse experiments, microglial CD39 and CD73-dependent adenosine generation formed a feedback pathway from extracellular ATP to neuronal A1 receptors, limiting neuronal activity [46]. Another study found that many cortical and thalamic synaptic properties could develop typically without microglia [51]. These results argue for specifying the circuit and developmental or disease state before assigning an obligatory pruning function.

TREM2 illustrates why glial metabolism cannot be interpreted as a uniformly detrimental increase. In amyloid models, TREM2–DAP12–SYK signaling supported metabolic adaptation through PI3K–AKT–GSK3β–mTOR, alongside SYK-independent responses [52]. A brain-transported TREM2 agonist enhanced microglial metabolic function in experimental Alzheimer systems [53]. CSF soluble TREM2 cannot be equated with signaling through the membrane receptor [50]. Nonetheless, they establish that restoring selected microglial functions and suppressing selected inflammatory outputs are different intervention goals.

## 4. Synaptic Plasticity and Cellular Energetics

### 4.1. Transmission Can Fail Before Synapses Disappear

**Figure 2:**
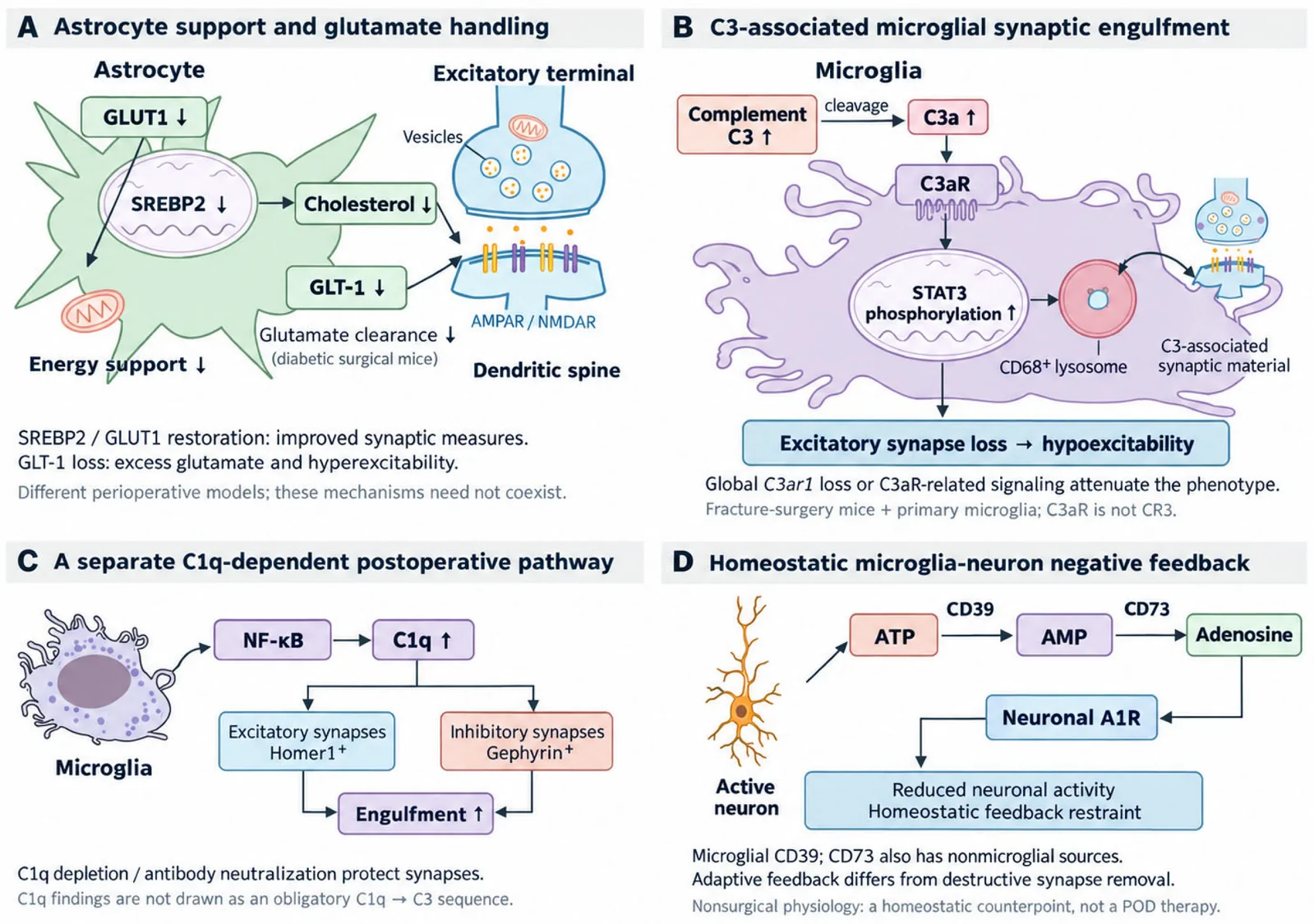
**Glial regulation of synaptic support, elimination, and activity**. (**A**) Separate surgical models implicate astrocytic cholesterol supply, glucose transport, and glutamate clearance [54,55,56]. (**B**) C3-associated synaptic changes, C3a–C3aR signaling, and downstream STAT3 phosphorylation are linked to microglial engulfment and excitatory synapse loss [41]. Global *C3ar1* knockout does not establish microglia-specific causality in vivo. (**C**) A separate C1q-dependent pathway involves engulfment of excitatory and inhibitory synaptic elements [42]. (**D**) Nonsurgical experiments demonstrate homeostatic microglia–neuron signaling through ATP-to-adenosine metabolism and neuronal A1 receptors [46]. C3a is the ligand for C3aR, which is distinct from CR3. The ADP intermediate is omitted for clarity. Colors distinguish the labeled cells and pathway components and do not encode quantitative values. Connecting arrows indicate the direction of signaling, metabolic conversion, or synaptic engulfment; upward and downward arrows indicate increases and decreases, respectively. The curved arrows in panel (**B**) depict synaptic engulfment.

Synaptic plasticity describes activity-dependent change, whereas basal transmission describes communication at a given moment. A reduced miniature excitatory postsynaptic current frequency, impaired long-term potentiation and reduced spine density therefore answer different questions. The first can reflect presynaptic release or functional synapse number; the second depends on induction and expression mechanisms; the third measures a structural correlate, as illustrated by the complementary physiological and anatomical endpoints in perioperative studies [41,54,55]. Combining these measures is stronger than treating any one as proof of a generalized loss of plasticity. Figure 2 separates the principal cellular processes and makes clear that much of the mechanistic evidence remains preclinical.

Glutamate signaling illustrates this distinction. A 2026 mouse study implicated enhanced interaction between TRPV4 and the GluN1 NMDA receptor subunit, associated with receptor phosphorylation, endoplasmic-reticulum stress and behavioral abnormalities. Pharmacological inhibition and region-specific *Trpv4* knockdown attenuated the phenotype [57]. The reported sequence was increased TRPV4–GluN1 interaction, higher GluN1 Ser896 phosphorylation and NMDA receptor activity, followed by ER-stress readouts involving PERK, IRE1α, ATF6 and CHOP [57]. These branches should not be mistaken for a fully resolved ordering of every ER-stress sensor. The study establishes a testable receptor-to-stress route in that model. It does not mean that global NMDA receptor blockade is a delirium treatment: the clinical effect depends on dose, circuit, state and the physiological roles of the receptor. The negative postoperative ketamine prevention trial provides a direct reminder that a molecularly plausible receptor intervention need not improve delirium [58].

Glutamate clearance can determine susceptibility to the same exposure. In diabetic mice, reduced astrocytic GLT-1 amplified surgery-associated neuronal hyperexcitability, and restoring GLT-1 or inhibiting hippocampal excitatory neurons improved postoperative cognition [56]. By contrast, other surgery models show reduced excitatory transmission after complement-associated synapse elimination [41]. These findings should not be flattened into a universal increase or decrease in excitation. Circuit dysfunction may result from inappropriate excitation, excessive inhibition or loss of coordinated input, and these states may differ across regions or over the course of one episode.

The relationship between inhibition and inflammation also extends beyond surgery. Experimental impairment of meningeal lymphatic function produced microglia-dependent IL-6 changes and altered inhibitory synaptic physiology; restoration of lymphatic function reversed age-associated abnormalities in mice [59]. Such findings support studying clearance and immune–circuit interactions, but they do not establish glymphatic or meningeal lymphatic failure as a demonstrated cause of human postoperative delirium. The transferable insight is that an immune pathway can regulate synaptic physiology without requiring widespread cell death.

### 4.2. Astrocytic Lipid and Glucose Support

Cholesterol handling is an especially relevant interface between APOE biology and synaptic function. A 2026 experimental study identified reduced astrocytic SREBP2, hippocampal cholesterol deficiency and impaired excitatory transmission after tibial-fracture surgery in aged mice. Astrocyte-specific SREBP2 overexpression or local cholesterol replenishment improved physiological and behavioral outcomes [54]. This supplies a concrete alternative to a purely inflammatory account of astrocyte involvement. However, associated blood 24-hydroxycholesterol changes in surgical patients are not equivalent to measurement of cholesterol availability at a human synapse, and cognitive decline in that cohort should not be recast as delirium.

Broader human biology supports coordinated neuronal and astrocytic maintenance: single-nucleus profiling of 191 prefrontal-cortex donors identified a shared program coupling neuronal synaptic expression to astrocytic cholesterol and synapse-related expression, which declined with age and in schizophrenia [60]. In developing human organoids, microglia transferred cholesterol-containing material and promoted neuronal maturation [61]. Together, these findings highlight the importance of intercellular lipid transfer and availability, rather than intracellular lipid accumulation alone [16,61].

Energy support is similarly cell-dependent. Overexpression of GLUT1 in hippocampal astrocytes improved dendritic-spine and plasticity measures in an aged-mouse postoperative cognitive model [55]. In contrast, a study of microglial GLUT1 associated increased glucose uptake with an inflammatory state and found benefit from microglia-targeted modulation [62]. The direction of a useful intervention therefore cannot be inferred from the transporter name alone. Suppressing glucose uptake globally could remove metabolic support from neurons or astrocytes while attempting to restrain immune-cell activation. Experiments should report the targeted population, the specificity of the genetic or viral strategy and the effect on both immune and neuronal metabolism.

These results also make a methodological point about brain imaging. Increased tissue glucose uptake could reflect activated glia despite inadequate neuronal energy supply, while a reduced signal could reflect medication effects or loss of neuronal activity. A tissue-level metabolic measure should be linked to cell-resolved assays before it is interpreted as improved or impaired synaptic energetics. Paired metabolic and electrophysiological measurements are particularly valuable because successful plasticity requires ion-gradient maintenance and activity-coupled substrate availability, not simply a high bulk concentration of a metabolite.

Metabolite delivery can additionally engage signaling pathways. In aged surgical mice, lactate supplementation improved cognitive and inflammatory outcomes alongside SIRT1-pathway changes; a SIRT1 inhibitor attenuated the protection [63]. This result does not prove that endogenous astrocyte-to-neuron lactate transfer is the deficient step. It separates a supplementation experiment from direct measurement of transport and flux, which would be required to establish that specific intercellular mechanism.

### 4.3. Mitochondria, Trophic Signaling and Chromatin

Mitochondrial mechanisms should likewise be specified by cell type and perturbation. In an experimental delirium model, astrocytic β-arrestin1 deficiency enhanced Drp1-associated mitochondrial fragmentation and inflammatory reactivity, whereas a β-arrestin1-biased intervention reduced abnormalities [64]. Other recent work links mitochondrial fission to postoperative delirium-like behavior under high-altitude deacclimatization [65]. The latter exposure is a specific interaction model, not evidence that mitochondrial fission is a universal mechanism in routine surgical patients. Pharmacological rescue is most persuasive when accompanied by genetic specificity and a demonstration that altered arousal or movement did not account for improved behavioral testing.

**Figure 3:**
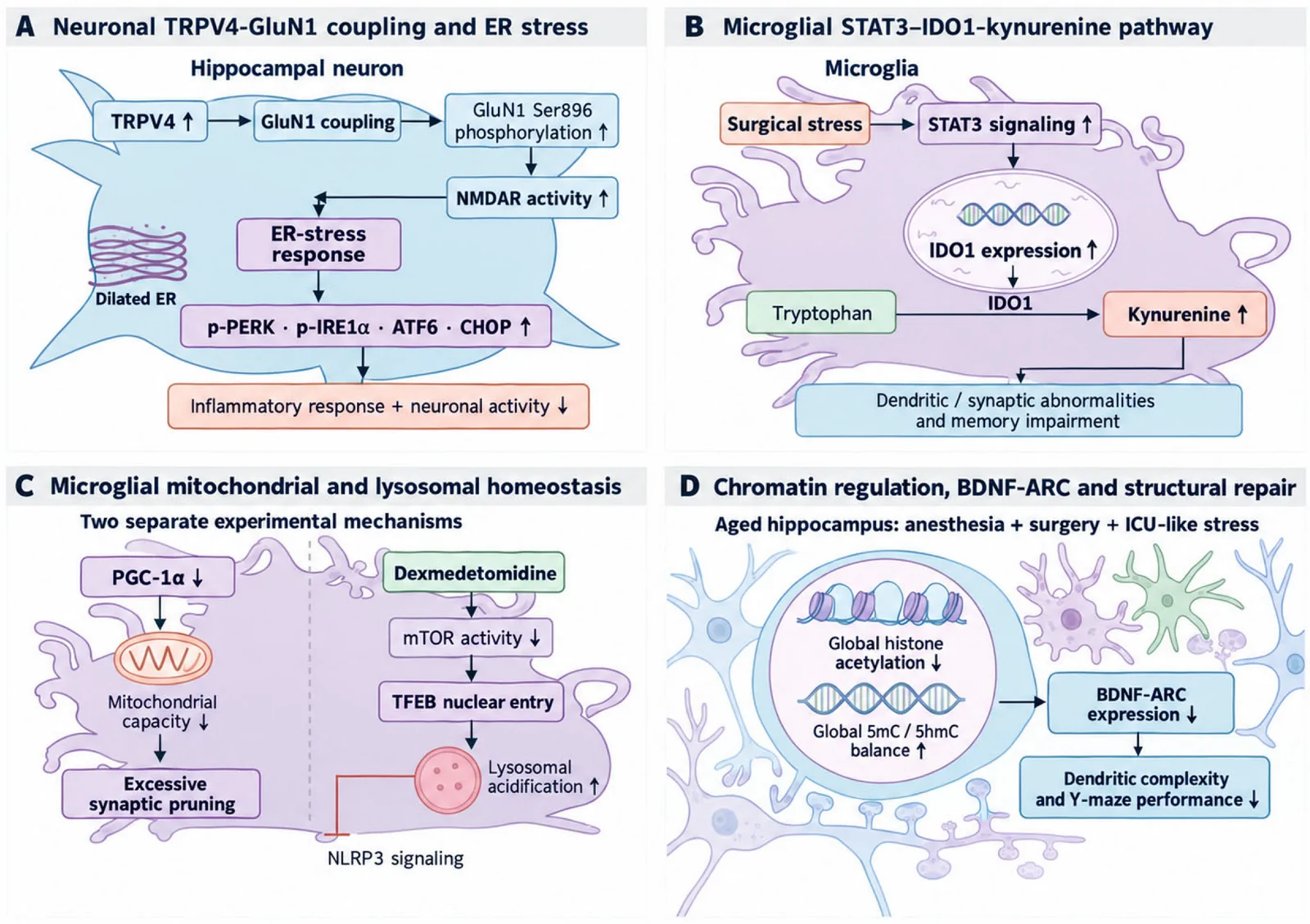
**Intracellular mechanisms linking perioperative exposures to synaptic impairment and recovery.** (**A**) Experimental TRPV4–GluN1 coupling, NMDA receptor activity, and ER-stress responses are implicated in postoperative neuronal dysfunction [57]. (**B**) Microglial STAT3–IDO1 signaling links kynurenine metabolism to synaptic abnormalities in aged fracture-surgery mice [68]. (**C**) Separate studies examine PGC-1α-dependent mitochondrial regulation and dexmedetomidine-associated mTOR–TFEB–lysosomal signaling [66,67]. (**D**) An aged anesthesia/surgery/ICU-stress model showed global hippocampal epigenetic alterations, reduced BDNF–ARC-related expression, dendritic abnormalities, and impaired Y-maze performance [25]. Global 5mC/5hmC measurements were not neuron-specific or locus-specific. The illustrated pathways represent experimental findings rather than established clinical treatments. ER, endoplasmic reticulum; TFEB, transcription factor EB. Colors distinguish the labeled cells, organelles, and pathway components and do not encode quantitative values. Connecting arrows indicate the proposed direction of signaling or downstream effects; upward and downward arrows indicate increases and decreases, respectively. The red blunt-ended line in panel (**C**) indicates inhibition of NLRP3 signaling. The dashed divider separates the two experimental mechanisms in panel (**C**). Neuronal processes extending beyond the panel boundaries are schematic continuations, not missing data.

Microglial mitochondrial regulation provides an additional link to synapse handling. In aged surgical mice, reduced PGC-1α accompanied mitochondrial dysfunction and excessive pruning; pharmacological activation and targeted overexpression improved synaptic and cognitive outcomes [66]. In a separate aged-mouse study, dexmedetomidine was associated with lower mTOR activity, greater TFEB nuclear localization, improved lysosomal acidification and reduced NLRP3-related inflammation; TFEB knockdown weakened protective effects in vitro [67]. The second chain remains a candidate explanation for an experimental drug response, rather than a mediator established in clinical dexmedetomidine trials. Figure 3 resolves these metabolic, receptor and chromatin mechanisms into separate cellular modules.

Trophic support offers another entry point. Astrocytic GDNF overexpression improved hippocampal plasticity and cognition after surgery in aged mice, with GFRα1/NCAM signaling implicated [69]. Exercise-conditioned plasma experiments connected cholinergic activity to BDNF/TrkB signaling and postoperative synaptic protection, using receptor antagonism and *Chrm1* knockdown to test dependence [70]. Together with BDNF–ARC epigenetic findings [25], these studies motivate analysis of impaired repair and activity-dependent transcription rather than inflammation alone. They do not show that a peripheral BDNF measurement captures central TrkB activity or that a nonspecific rise in a trophic factor constitutes target engagement.

Recent adult-circuit work identifies astrocytic CCN1 as another maintenance signal: loss of this secreted factor destabilized visual-cortical circuitry, with effects on inhibitory-neuron maturation and oligodendrocyte differentiation [71]. This expands the range of candidate recovery pathways without demonstrating a perioperative role. Conversely, a cardiac-surgery study of 107 analyzed patients found no independent association of plasma BDNF or the Val66Met variant with POD [72]. Positive trophic-factor rescue in a mouse and a negative peripheral human association address different biological questions, and both belong in a balanced account.

A credible mechanistic chain should contain a defined upstream exposure, a cell-specific regulatory change, a synaptic physiological effect and rescue of an appropriate behavioral domain. For example, restoring a trophic pathway should recover a measurable plasticity deficit while preserving normal inhibitory control. Single-cell and spatial studies of nonsurgical memory formation demonstrate that neuron–astrocyte transcriptional relationships can persist and influence memory expression [73]. Applying this resolution to perioperative models could distinguish reduced capacity for recovery from an acute change in neurotransmission. Table 2 compares pathways for which the experimental evidence is informative and identifies what is still missing in humans.

### 4.4. Neuroactive Metabolites Connect Immune Activity to Receptors

The kynurenine pathway provides unusually direct human evidence for an immune–metabolic connection. In a multicenter study of 586 hospitalized patients, including 248 with delirium, CSF quinolinic acid was associated with delirium, neurofilament light and subsequent mortality, with associations persisting after adjustment [74]. Because the cohort included acute hip fracture and delirium around the surgical period, these findings are relevant to perioperative vulnerability but do not isolate the effect of anesthesia. They support studying neuroactive metabolites in the central compartment rather than assuming that a serum measurement represents the local exposure of neurons.

A 2026 aged-mouse fracture study sharpened the upstream sequence: surgery induced microglial STAT3-related IDO1 expression, shifted tryptophan metabolism toward kynurenine and accompanied dendritic and synaptic abnormalities [68]. IDO1 inhibition was protective, while exogenous kynurenine reproduced impairment; microglia-targeted IDO1 overexpression weakened berberine-associated rescue [68]. This is a more specific perturbation chain than a bulk inflammatory correlation. However, a rise in kynurenine is not itself proof of increased quinolinic acid at neuronal receptors, so the experimental IDO1 result and the human CSF quinolinic-acid association should remain linked hypotheses rather than one completed causal demonstration [68,74].

Mechanistically, the relevant question is whether metabolite changes alter receptor function at concentrations achieved in the brain, and whether they precede or follow the clinical episode. A correlation with neurofilament can reflect a shared inflammatory injury process rather than prove that one metabolite produced the injury. Interventions directed at pathway enzymes could also redistribute substrate into other branches, making measurement of several metabolites more informative than measurement of a single product. In a translational experiment, biochemical target engagement should therefore be accompanied by electrophysiology and a behavioral measure of attention, with controls for sickness and reduced movement. This design would directly test whether modifying the pathway restores neural function rather than merely changing a biomarker associated with severe illness.

Evidence derives primarily from postoperative animal models, supplemented by cell-based experiments and genetic or pharmacological interventions. Human observational findings are summarized separately in Table 1, while nonsurgical models provide indirect mechanistic context. Experimental findings support mechanistic plausibility but do not establish clinical efficacy in human postoperative delirium. C3aR signaling is distinct from CR3-mediated opsonophagocytosis.

## 5. Human Synaptic Markers, Network Dysfunction and Recovery

### 5.1. Molecular Markers Do Not All Support the Same Interpretation

Direct evidence of synaptic structural injury during human delirium remains limited. In a multicenter hip-fracture study of 392 patients, CSF neurogranin did not differ significantly between patients with and without delirium, including after adjustment for relevant covariates [26]. This is important negative evidence against using that marker as a general synaptic signature of delirium. It does not exclude altered receptor trafficking, presynaptic release or circuit-specific dysfunction that might occur without a detectable neurogranin change. Nor can a sample collected at one time point establish whether a brief earlier or later change occurred.

Neuroaxonal and glial markers provide complementary, rather than interchangeable, information. A perioperative matched study associated plasma neurofilament light with delirium risk, severity and subsequent cognitive change [75]. However, a larger recent hip-fracture analysis found that the overall association between CSF neurofilament light and delirium was attenuated after adjustment and persisted mainly in patients without pre-existing dementia [76]. These differences highlight the importance of background disease, renal function, compartment and sampling time. Elevated neurofilament cannot by itself establish that an acute episode caused new synaptic loss.

Plasma tau changes have been associated with delirium incidence and severity and can decline as symptoms improve [77]. Yet mouse work indicates that perioperative phosphorylated-tau changes may involve peripheral production and immune-cell biology [78]. This does not negate their clinical value, but it cautions against assuming a uniquely neuronal source. Human CSF amyloid and tau profiles also associate with delirium in some patients without diagnosed dementia, supporting latent Alzheimer-related pathology as one susceptibility context [79]. A marker of pre-existing vulnerability must be distinguished from a marker of the current episode or its consequences.

Serial sampling helps make that distinction: a prospective surgical study obtained inflammatory and neuronal-injury markers before surgery and at several early postoperative intervals, with later cognitive follow-up [37]. Its IL-6 and phosphorylated-tau findings reinforce the importance of trajectories, but the modest cohort size and multiple markers limit definitive subtype assignment. A biomarker panel should consequently specify the sampling window as part of its definition, rather than treating every postoperative value as interchangeable [37,75].

Proteomic and metabolomic panels can identify combinations that are more informative than individual markers. A small preoperative SOMAmer study reported substantial apparent discrimination that fell when evaluated by leave-one-out cross-validation [80]. Recent CSF polyamine work identified associations involving neurotransmitter and nitrogen metabolism [81]. These are useful discovery signals, but model size, selection procedures and validation strategy determine whether performance will persist. A clinically useful panel needs independent validation against a strong clinical baseline, appropriate handling of missing data and evaluation at the actual time when its result would influence care [80,81,82].

The same caution applies to central proteomics. An aptamer-based study of 24 matched case–control pairs found nominal preoperative CSF protein differences and pathway signals, but the individual proteins did not remain significant after multiple-testing correction [83]. Such a result should be reported as a discovery study rather than a validated synaptic fingerprint. Shared use of an underlying cohort across publications can also make apparent replication misleading. A reference to several papers is not equivalent to several independent patient populations; cohort provenance and overlapping samples should be checked when evidence is synthesized.

### 5.2. Electrophysiology Connects Cell Function to the Syndrome

Electroencephalography provides a temporally accessible measure of brain function. Post-cardiac-surgery delirium has been associated with reduced alpha-band connectivity and altered directionality of information flow [84]. Resting-state functional MRI has also identified reduced network integration during delirium, although motion-free patient samples were small [85]. These observations support a disorder of distributed communication. They do not identify a unique receptor abnormality or establish that the same network pattern occurs in every motor subtype or clinical context.

Recent studies separate baseline vulnerability from acute dysfunction more explicitly. Lower anesthetic-dose-adjusted alpha power was associated with delirium and poorer preoperative processing speed in pooled prospective data [86]. In a 2026 study, reduced preoperative connectivity within attention, cognitive-control and default-mode networks characterized patients who subsequently developed delirium [87]. Such associations suggest that anesthesia and surgery may reveal limited reserve, but they are not sufficient to classify an individual patient without externally validated thresholds and an understanding of medication effects.

A high-density EEG cohort found postoperative slowing of peak alpha frequency and a steeper spectral exponent, with larger changes in patients with delirium [88]. These features are compatible with altered excitatory–inhibitory physiology, but spectral exponent is not a direct measurement of synaptic inhibition. A separate multicenter cohort found lower preoperative beta amplitude-based connectivity in patients who later developed delirium, while detecting no persistent group-level quantitative EEG differences at three months [27]. Because EEG was recorded only before surgery and at three months, these findings do not establish the trajectory of electrophysiological abnormalities during acute delirium or their subsequent resolution.

Signal complexity offers a complementary description. Perioperative EEG analyses have associated lower Lempel–Ziv complexity with more severe delirium, including effects not fully explained by measured peripheral inflammation [89]. Other studies examined spectral responses during propofol induction [90] or combined preoperative occipital alpha power with cognitive assessment in a small independent validation cohort [91]. These approaches interrogate different states and should not be combined into one biomarker without harmonization. An induction response may reveal sensitivity to a pharmacological challenge, while an awake recording measures baseline or acute network function. Repeated measurements under comparable conditions are needed to determine which signal is a trait and which is a state.

### 5.3. Matching Time Scales to Biological Claims

Rapid fluctuations favor mechanisms capable of changing within minutes or hours: neuromodulation, receptor function, metabolic availability and network coordination [44,46,57,88]. Structural synapse removal, myelin remodeling and stable chromatin changes operate on different schedules, although initiation can occur early. This temporal argument is a mechanistic interpretation, not proof that structural injury is absent. It predicts that some patients may improve clinically while molecular evidence of injury persists, whereas others may show reversible physiological disruption with little measurable structural injury.

Recovery studies should therefore distinguish resolution of diagnostic criteria, restoration of attention, return of functional independence and return to the preoperative cognitive trajectory. Baseline cognition and an appropriate comparison group are necessary to interpret later decline [2,27,37]. An exploratory ICU methylation study linked longer-term cognitive outcomes to immune-related signals, including a significant CpG in the major histocompatibility complex region [92]. Because that population differs from elective surgical patients, this finding is best used to motivate longitudinal testing rather than define a postoperative mechanism. Molecular persistence, physiological persistence and clinical persistence should be measured rather than assumed to coincide.

## 6. Translation into Prevention and Mechanistic Trials

Collectively, human and experimental studies provide convergent evidence implicating *APOE*-related susceptibility, neuroimmune and complement signaling, and glial–metabolic responses in perioperative brain dysfunction [4,5,6,9,13,41,68,74]. Nevertheless, convergence at the pathway level does not establish identical causal mechanisms in humans and experimental models. Complement-dependent synaptic elimination, astrocytic metabolic support, and BDNF–ARC regulation have primarily been investigated using postoperative memory outcomes in animals [25,41,54,55]. Human electrophysiological studies provide complementary evidence of network dysfunction [84,88,89], but direct links between individual molecular pathways and impaired attention or arousal remain to be established.

### 6.1. Established Clinical Questions Constrain Mechanistic Claims

Preventive care can improve outcomes without identifying one molecular target. A randomized, tailored, family-involved Hospital Elder Life Program reduced postoperative delirium in a noncardiac surgical population [93]. Its components act across orientation, mobility, sleep and other modifiable vulnerabilities. Such findings are compatible with improved circuit resilience, but they do not prove that a particular synaptic pathway mediates the benefit. Molecular studies nested within effective interventions would be more informative than retrospective assignment of a favored mechanism to a complex clinical bundle.

A larger stepped-wedge trial involving 1470 older patients also supported a multicomponent prevention program, with benefit in orthopedic or abdominal surgery but not a demonstrated benefit in the cardiac subgroup [94]. Preoperative cognitive training reduced delirium in a smaller coronary-bypass trial, although the investigators considered the result exploratory because of the limited number of events [95]. These interventions are relevant to activity-dependent plasticity, but improved reserve remains an explanation to test. Training effects, social engagement, expectation, adherence and perioperative care changes can coexist. Trials incorporating baseline and postoperative physiology could determine whether benefit tracks a reproducible change in the functional response to surgery.

Anesthesia trials further constrain simple causal stories. Regional anesthesia without sedation did not reduce delirium relative to general anesthesia in the RAGA trial [96]. ENGAGES-Canada reduced anesthetic exposure and EEG suppression without reducing postoperative delirium [97]. A 2026 trial comparing total intravenous and volatile anesthesia also reported similar delirium findings on its day-3 assessment, an endpoint that may miss episodes outside that window [98]. These studies do not mean that anesthetic exposure is irrelevant. They show that changing one exposure label or physiological surrogate does not reliably remove the multiple determinants of delirium.

### 6.2. Pharmacological Interventions Require Timing and Target Engagement

Mechanistic plausibility alone does not establish clinical efficacy; dexmedetomidine trials illustrate the importance of regimen and clinical setting. In DECADE, an infusion beginning before cardiac surgery and continuing for 24 h did not reduce delirium [99]. In MINDDS, a brief nighttime dose in selected, early-extubated cardiac-surgery patients reduced delirium on postoperative day 1, while the difference over three days was not statistically significant [100]. These trials should be read together. Sedation, sleep promotion, sympatholysis, analgesic sparing and immunomodulation may all contribute, and the favorable effect of one schedule does not validate a universal microglial mechanism or all dosing strategies.

An earlier trial in 700 older patients admitted to intensive care after noncardiac surgery found lower delirium incidence with a low-dose overnight dexmedetomidine infusion [101]. Its contrast with DECADE reinforces that the population, starting time, dose and outcome window form part of the intervention itself. Pooling across regimens may estimate an average effect while obscuring which clinical setting is most likely to benefit. For a mechanistic substudy, the important measurements would include achieved exposure, arousal during assessment, sleep-related physiology and the proposed immune mediator, rather than a single postoperative cytokine concentration.

The APOE mimetic CN-105 provides a closer link between genetic biology and intervention. The phase 2 MARBLE trial randomized 186 older surgical patients who underwent surgery, with safety and feasibility as its primary focus. Delirium occurred in 19.3% of CN-105 recipients and 26.5% of placebo recipients; the odds ratio was 0.66 with a 95% confidence interval of 0.31–1.42 [102]. The study supports further testing, but does not establish delirium prevention. A larger trial should prespecify the clinical endpoint, exposure window and molecular target-engagement measures; any genotype–treatment interaction needs its own adequately powered analysis.

Other receptor-based interventions further illustrate these constraints. In the PODCAST trial, intraoperative ketamine did not prevent delirium in older adults undergoing major surgery [58]. Rivastigmine, tested as an adjunct for delirium during critical illness, did not improve the clinical course and the trial was stopped early because of concerning outcomes [103]. These populations and interventions differ, but both caution against translating a neurotransmitter-deficiency or receptor-signaling hypothesis directly into treatment. Prevention, treatment of an established episode and acceleration of cognitive recovery are separate indications.

Medication burden supplies a complementary observational signal. A large, nested case–control study associated higher preoperative anticholinergic burden with POD [104]. This supports careful measurement of cumulative receptor-active exposures in mechanistic cohorts; residual confounding and drug indications prevent a causal effect estimate from that association alone. It also does not imply that administering a cholinesterase inhibitor reverses the risk, given the different timing, populations and results of the rivastigmine trial [103,104]. Table 3 places selected trials alongside the mechanistic conclusions they can and cannot support.

### 6.3. A Practical Design for Causal Validation

The next generation of studies should connect measurements across levels in the same individuals. Before surgery, genotype, cognitive phenotype, frailty and a baseline blood or EEG measurement define susceptibility. During the perioperative period, repeated assessments of attention and arousal should be aligned with molecular sampling, medication exposure and complications. Follow-up then determines whether the molecular and physiological signals resolve, persist or predict a changed trajectory. The cellular mechanisms in Figure 3 identify candidate measurements for this study sequence. It avoids interpreting a postoperative marker as a preoperative predictor or a static baseline characteristic as evidence of acute injury.

Phenotyping requires particular care in hip-fracture and critical-care populations, where delirium may precede surgery or sampling. Prevalent and incident delirium should be separated, and sedation or coma should not be coded as a negative delirium assessment. Repeated structured assessments are preferable to a single postoperative screen, while discharge and death require explicit handling because they truncate observation. Molecular analyses should account for assay batch, blood cell composition, organ function and exposure timing. If a classifier is developed, patient-level data partitioning must precede feature selection to prevent optimistic performance estimates.

Prediction studies must also declare their intended decision time. The BioCog algorithm combined preoperative information, procedural characteristics and postoperative laboratory changes, with internal validation in 929 participants [82]. This is an early-postoperative risk-evaluation strategy, not a purely preoperative test. A new molecular panel should be compared with an appropriately timed clinical model and evaluated for calibration, net benefit and performance in a separate institution. Improvement in discrimination alone does not demonstrate better care, and the intervention triggered by a positive result should be specified before clinical implementation.

Experimental systems should be chosen to test a defined part of the chain. A human-cell blood–brain barrier-on-chip model has already been combined with a mouse perioperative study to examine endothelial, pericyte and immune interactions [105]. Similar platforms could test whether *APOE* background changes the response to a defined inflammatory exposure. Induced pluripotent stem cell-derived neurons, astrocytes and microglia would permit cell-specific perturbation of complement regulation or lipid support, but their developmental state and lack of systemic physiology remain limitations. In animals, attention, arousal, motor behavior, pain and sleep should be assessed alongside memory, with anesthesia-only and surgery-related controls where feasible.

The decisive experiment is not simply a reduction in an inflammatory protein. A proposed target should show engagement in the intended compartment, restoration of a relevant synaptic or network abnormality and improvement in delirium burden without impairing infection control, hemodynamics or recovery. Mechanistic rescue should be reproduced with complementary approaches, especially when a drug has several known actions. For complement and chromatin targets, transient or cell-specific modulation may be more interpretable than prolonged systemic inhibition. These are priorities for testing, not recommendations for current clinical prescribing. Interventions initiated before surgery or before documented behavioral impairment should be interpreted as preventive or perioperative protective strategies rather than evidence of reversal of established deficits.

## 7. Limitations

This narrative review has several limitations. First, the targeted literature search was not systematic, and no formal risk-of-bias assessment or quantitative synthesis was performed, leaving the possibility of selection and publication bias. Second, the included studies varied in patient populations, delirium definitions, sampling times, biological compartments, and experimental endpoints, limiting direct comparisons. Third, much of the mechanistic evidence derives from animal models of postoperative cognitive impairment or nonsurgical conditions rather than clinically confirmed human delirium. Accordingly, the proposed mechanistic relationships should be considered hypothesis-generating rather than established causal pathways in patients.

## 8. Conclusions

Recent genetics and molecular profiling have made perioperative delirium a more tractable biological problem. *APOE*-associated susceptibility, complement-regulatory signatures and human glial transcriptional differences provide anchors for experimental work. The most concrete synaptic mechanisms currently come from models that manipulate complement, astrocytic metabolic support, receptor signaling or activity-dependent transcription. Their relevance to human delirium depends on the phenotype, cell type, anatomical circuit and time window being studied. Negative neurogranin findings, context-dependent injury biomarkers, and the absence of persistent group-level EEG differences at three months argue against a single obligatory pathway from inflammation to irreversible synapse loss. Progress will depend on linking inherited risk and dynamic cell states to measured synaptic physiology, then demonstrating that an intervention improves attention, delirium burden and recovery in a well-defined patient population. At present, no individual molecular pathway has been sufficiently validated to guide routine biomarker-based assessment or pathway-targeted therapy for postoperative delirium.

## Figures and Tables

**Table 1:** Selected human evidence connecting genetic susceptibility, molecular state and perioperative delirium.

Study and Population	Design and Sample	Main Finding	Interpretive Limit
Armstrong et al., 2026 [4]	UK Biobank surgical GWAS; 1016 cases and 139,148 controls. POD identified using ICD-10 F05 codes; genetic association study.	*APOE*-region association; adjusted odds ratios of 1.75 and 4.19 for one and two ε4 copies.	Coded outcome; linked genes at one region are not independent causal discoveries.
Raptis et al., 2026 [5]	All-cause delirium GWAS meta-analysis; 11,931 cases within 1,059,130 participants.	Strong *APOE* signal; integration with proteomics and Alzheimer-related genetic analyses.	Not confined to surgery; multi-trait results require phenotype-specific validation.
Surgical biobank study, 2026 [15]	33,526 surgical patients; predominantly European ancestry.	*APOE* ε4 associated with delirium and other neurocognitive diagnoses.	Ascertainment and population differ from bedside-adjudicated perioperative cohorts.
MINDDS transcriptomics, 2025 [6]	RNA sequencing in 51 participants; *C4BPA* PCR analysis in 319. Twice-daily CAM assessments through postoperative day 3; blood collected preoperatively and on day 1.	Lower preoperative *C4BPA* and *CD55* expression associated with delirium.	Blood signature does not establish central complement activation or synapse loss.
Perioperative expression cohort, 2026 [7]	599 older patients; baseline and postoperative blood expression. Delirium monitored through postoperative day 7; blood collected preoperatively and on day 1.	Postoperative immune-related expression differences; no significant preoperative transcriptome-wide differences.	Cell composition and concurrent systemic illness can influence expression.
Ishii et al., 2026 [8]	Epilepsy resection tissue; 18 patients, nine subsequently developing POD. Brain tissue obtained during epilepsy surgery; postoperative delirium classified retrospectively.	Microglial inflammatory and astrocytic pathway signatures.	Small heterogeneous surgical sample; no genome-wide significant methylation sites.
Methylation cohorts, 2024–2026 [20,24]	Four-cohort replication; separate hip-fracture study of 65 selected patients. CAM-ICU, DRS-R-98, or chart adjudication; blood collected preoperatively and on day 0 or day 3.	Replicated candidate CpGs and time-dependent immune-pathway enrichment.	Association and enrichment do not establish causal regulation in brain cells.
CSF neurogranin, 2025 [26]	Multicenter hip-fracture study; 392 patients. Daily DSM-5 delirium assessments; CSF collected during spinal anesthesia before anesthetic administration.	No significant association between neurogranin concentration and delirium.	One synaptic marker and sampling window cannot exclude all functional synaptic changes.
Longitudinal EEG, 2026 [27]	330 surgical patients with usable EEG; 59 POD cases; 57 nonsurgical controls. POD assessed through day 7; EEG recorded preoperatively and at three months.	Lower preoperative beta connectivity; no significant POD-related differences in EEG changes from baseline to three months.	No EEG recordings during delirium; acute electrophysiological changes and their recovery cannot be directly assessed.

GWAS, genome-wide association study; POD, postoperative delirium; CSF, cerebrospinal fluid; EEG, electroencephalography. Sample sizes are study-specific and should not be summed because cohort overlap may occur. Publication year follows the indexed journal issue where available.

**Table 2:** Experimental synaptic mechanisms and the evidence still needed for human delirium.

Pathway	Experimental Evidence	Synaptic Implication	Unresolved Human Question
C3–C3aR–STAT3 [41]	Fracture-surgery mice; global *C3ar1* knockout and pharmacological C3aR/STAT3 inhibition improved synaptic and memory outcomes. Microglial involvement was supported separately in cell-based assays. Y-maze and location memory tests; dendritic spines, hippocampal LTP, and microglial VGluT1 engulfment.	Excitatory synapse elimination and neuronal hypoexcitability.	Does central pathway activity precede a clinically defined episode?
C1q and microglial engulfment [42]	Microglial C1q depletion or neutralization attenuated postoperative synaptic and memory abnormalities. Y-maze and trace fear conditioning; Homer1/gephyrin colocalization, microglial engulfment, and Golgi analysis.	Both excitatory and inhibitory synaptic elements can be affected.	Which synapse populations are involved, and at which time points?
Peripheral complement sources [13,14]	Liver-derived C3 and IL-17/C/EBPβ-dependent pathways tested in surgical models.	Systemic immune processes may influence local synaptic remodeling.	How much central complement activity is peripheral in origin?
Astrocytic SREBP2 and lipids [54]	Aged mice; astrocytic SREBP2 restoration or local cholesterol replenishment improved outcomes. Y-maze and object-recognition tasks; hippocampal LTP and excitatory synaptic transmission.	Cholesterol availability supports plasticity and excitatory transmission.	Do patient lipid markers reflect local synaptic supply?
Glutamate clearance and receptors [56,57]	GLT-1 restoration in diabetic mice; TRPV4 inhibition or knockdown in another model.	Different perturbations produce hyperexcitability or receptor-associated stress.	Which receptor and circuit state characterize each patient subgroup?
Cell-specific glucose transport [55,62]	Astrocytic GLUT1 support and microglial Glut1 modulation showed different beneficial directions.	Neuronal support and immune-cell metabolism need separate measurements.	Can an intervention target one compartment without impairing another?
BDNF–ARC and trophic support [25,69,70]	Chromatin, GDNF and cholinergic/TrkB perturbations improved selected animal outcomes. Y-maze performance; Golgi-based dendritic length, branching, and spine density.	Activity-dependent transcription may contribute to plasticity and repair.	Is a specific regulatory change causal and reversible in human POD?
Mitochondrial dynamics [64,65]	Astrocytic β-arrestin1/Drp1 and context-specific fission mechanisms examined in mice.	Energetic and inflammatory changes can impair synaptic performance.	Which cell type and exposure explain mitochondrial abnormalities clinically?
Microglial STAT3–IDO1 [68]	Aged fracture-surgery mice; IDO1 inhibition, kynurenine challenge and targeted IDO1 overexpression. Morris water maze and novel object recognition; dendritic morphology and synaptic protein measurements.	Tryptophan–kynurenine metabolism linked to dendritic and synaptic abnormalities.	Does central IDO1 activity precede POD, and which downstream metabolite is causal?
PGC-1α and TFEB [66,67]	PGC-1α activation/overexpression rescued pruning; TFEB knockdown tested a separate dexmedetomidine mechanism.	Mitochondrial capacity and lysosomal function are distinct glial targets.	Can beneficial homeostasis be restored without broadly suppressing microglia?
SETD3–NLRP3 [12]	Surgery model and cell experiments; SETD3 knockdown and promoter-associated H3K4 methylation assays.	Inflammatory transcription offers an upstream route to circuit dysfunction.	Is the same cell-specific chromatin mechanism active during human POD?

**Table 3:** Selected clinical trials and their implications for mechanism-based prevention.

Intervention and Trial	Population and Endpoint	Key Result	Mechanistic Interpretation
Tailored family-involved HELP [93]	281 older noncardiac surgical patients; in-hospital POD.	Delirium: 2.6% versus 19.4%; RR 0.14 (95% CI 0.05–0.38).	Supports multicomponent prevention; no single molecular mediator established.
Multicenter prevention program [94]	1470 older elective surgical patients; stepped-wedge cluster design.	Overall OR 0.87 (95% CI 0.77–0.98); subgroup benefit not demonstrated in cardiac surgery.	Population and delivery context influence effects.
Regional versus general anesthesia; RAGA [96]	950 randomized hip-fracture patients; seven-day POD.	Delirium: 6.2% versus 5.1%; risk difference 1.1 percentage points (95% CI −1.7 to 3.8).	Avoiding general anesthesia alone did not prevent delirium.
EEG-guided anesthesia; ENGAGES-Canada [97]	1140 randomized older cardiac-surgery patients; POD days 1–5.	Delirium: 18.15% versus 18.10%, despite less EEG suppression.	Changing an associated physiological surrogate did not establish clinical benefit.
Perioperative dexmedetomidine; DECADE [99]	794 analyzed cardiac-surgery patients; infusion through 24 h.	Delirium: 17% versus 12%; RR 1.48 (97.8% CI 0.99–2.23).	No prevention benefit for this regimen; findings cannot be generalized to all schedules.
Nighttime dexmedetomidine; MINDDS [100]	394 modified-intention-to-treat participants; primary endpoint on day 1.	Among assessed patients: 2.9% versus 8.5%; OR 0.32 (95% CI 0.10–0.83). Three-day difference not significant.	Timing, extubation selection and endpoint window matter.
APOE mimetic CN-105; MARBLE [102]	186 randomized surgical patients; phase 2 safety and feasibility focus.	Delirium: 19.3% versus 26.5%; OR 0.66 (95% CI 0.31–1.42).	Feasibility supports further study; efficacy remains unproven.
Ketamine; PODCAST [58]	Older adults undergoing major surgery; delirium prevention.	No reduction in postoperative delirium.	NMDA-related biological plausibility does not establish preventive efficacy.

Comparisons are intervention versus control within each trial. Trials differ in surgery, ascertainment, regimen and follow-up; percentages are not intended for indirect ranking. CI, confidence interval; OR, odds ratio; RR, risk ratio; HELP, Hospital Elder Life Program. This table is a selected narrative comparison, not a meta-analysis.

## Data Availability

No new experimental or participant-level data were generated or analyzed. All evidence discussed is available in the cited publications.

## Abbreviations

APOE, apolipoprotein E; BDNF, brain-derived neurotrophic factor; CAM, Confusion Assessment Method; CSF, cerebrospinal fluid; EEG, electroencephalography; GWAS, genome-wide association study; ICU, intensive care unit; IL, interleukin; NMDA, N-methyl-D-aspartate; POD, postoperative delirium; SREBP2, sterol regulatory element-binding protein 2; TrkB, tropomyosin receptor kinase B.
